# A test-retest fMRI dataset for motor, language and spatial attention functions

**DOI:** 10.1186/2047-217X-2-6

**Published:** 2013-04-29

**Authors:** Krzysztof J Gorgolewski, Amos Storkey, Mark E Bastin, Ian R Whittle, Joanna M Wardlaw, Cyril R Pernet

**Affiliations:** 1 Institute for Adaptive and Neural Computation, University of Edinburgh, 10 Crichton Street, Edinburgh, EH8 9AB, UK; 2 Brain Research Imaging Centre, a SINAPSE Collaboration centre, University of Edinburgh, Western General Hospital, Crewe Road, Edinburgh, EH4 2XU, UK; 3 Division of Clinical Neurosciences, University of Edinburgh, Western General Hospital, Crewe Road, Edinburgh, EH4 2XU, UK

**Keywords:** Test-retest, Overt verb generation, Covert verb generation, Overt word repetition, Landmark, Motor, fMRI, DTI

## Abstract

**Background:**

Since its inception over twenty years ago, functional magnetic resonance imaging (fMRI) has been used in numerous studies probing neural underpinnings of human cognition. However, the between session variance of many tasks used in fMRI remains understudied. Such information is especially important in context of clinical applications. A test-retest dataset was acquired to validate fMRI tasks used in pre-surgical planning. In particular, five task-related fMRI time series (finger, foot and lip movement, overt verb generation, covert verb generation, overt word repetition, and landmark tasks) were used to investigate which protocols gave reliable single-subject results. Ten healthy participants in their fifties were scanned twice using an identical protocol 2–3 days apart. In addition to the fMRI sessions**,** high-angular resolution diffusion tensor MRI (DTI), and high-resolution 3D T1-weighted volume scans were acquired.

**Findings:**

Reliability analyses of fMRI data showed that the motor and language tasks were reliable at the subject level while the landmark task was not, despite all paradigms showing expected activations at the group level. In addition, differences in reliability were found to be mostly related to the tasks themselves while task-by-motion interaction was the major confounding factor.

**Conclusions:**

Together, this dataset provides a unique opportunity to investigate the reliability of different fMRI tasks, as well as methods and algorithms used to analyze, de-noise and combine fMRI, DTI and structural T1-weighted volume data.

## Data description

### Original purpose of the data acquisition

The following dataset was acquired to validate fMRI tasks used in pre-surgical planning for tumor resection. Estimation of between session variance of cortical mapping is crucial for choosing tasks that provide surgeons with reliable information leading to safer procedures. Findings from this investigation were reported in [1]. Additionally this data was also used to compare single-subject fMRI statistical thresholding techniques [2].

### Participants and procedure

Eleven healthy volunteers over 50 years of age were recruited to match the mean age of diagnosis of a group of brain tumor patients undergoing resection surgery [3]. Data from one participant were discarded due to problems with executing the tasks. The remaining 10 subjects (median age 52.5 years, min=50, max=58) included four males and six females, of which three were left–handed and seven right–handed. Each subject was scanned twice, either 2 (eight subjects) or 3 (two subjects) days apart. The study was approved by South East Scotland Research Ethics Committee 01. All subjects were informed that the data collected during this study may be shared with other researchers given that the data would be anonymized, (and a template consent form is included in the data release).

### Behavioural tasks

Participants performed five behavioral tasks (Table 1): overt word repetition, covert verb generation, overt verb generation, motor movements, and landmark. The first three tasks were aimed at mapping language areas of the brain with (overt) or without (covert) actual speech production. To monitor each subject’s performance during the overt tasks, a sparse sampling technique was employed so staff could hear the subjects speaking [4]. The motor task consisted of finger tapping, foot twitching and lip poaching interleaved with fixation at a cross. Finally, the landmark task was designed to mimic the line bisection task used in neurological practice to diagnose spatial hemineglect [5]. Two conditions were contrasted, specifically judging if a horizontal line had been bisected exactly in the middle, versus judging if a horizontal line was bisected at all.

**Table 1:** **Summary of the MR parameters according to the guidelines published at**http:// http://practicalfmri.blogspot.de/2013/01/a-checklist-for-fmri-acquisition.html

**Sequence**	**T1**	**DTI**	**fMRI**				
			**Overt word repetition**	**Covert verb generation**	**Overt verb generation**	**Motor**	**Landmark**
**Manufacturer**	GE						
**Scanner model**	Signa HDxt						
**Magnetic field strength**	1.5T						
**Rx coil type**	8 channel phased-array						
**Pulse sequence type**	3D IRP (inversion recovery prepared)	single-shot spin-echo echo-planar imaging	single-shot gradient-echo echo-planar imaging				
**Number of shots (if > 1)**	n/a	n/a	n/a				
**PE acceleration factor (if > 1)**	n/a	n/a	n/a				
**PE acceleration type (if > 1)**	n/a	n/a	n/a				
**PE partial Fourier scheme (if used)**	n/a	n/a	n/a				
**In-plane FOV**	256 × 256 mm	256 × 256 mm	256 × 256 mm				
**In-plane matrix**	256 × 256	128 × 128	64 × 64				
**In-plane inline filtering (if any)**	n/a	n/a	n/a				
**Slice thickness**	1.3 mm	2 mm	4 mm				
**Inter-slice gap**	0 mm	0 mm	0 mm				
**Number of slices**	156	72	30				
**Slice acquisition order**	front to back	top to bottom	interleaved				
**Slice orientation**	coronal	axial	axial				
**TR**	10 s	16.5 s	5 s	2.5 s	5 s	2.5 s	2.5 s
**TE**	4 s	98 ms	50 ms				
**No. of volumes in time series**	1	71 (7 × b = 0 and 64 × b = 1000 s/mm2)	76	173	88	184	238
**No. of averages/volume (if > 1)**	n/a	n/a	n/a				
**Excitation flip angle (deg)**	8	90	90				
**Fat suppression scheme**	n/a	n/a	n/a				
**Sparse sampling delay (if used)**	n/a	n/a	2.5 s	n/a	2.5 s	n/a	n/a
**Prospective motion correction scheme (if used)**	n/a	n/a	n/a				
**Cardiac gating (if used)**	n/a	n/a	n/a				

Behavioural paradigms were implemented using Presentation® Software (Neurobehavioral Systems, Inc., USA). Stimuli synchronisation and presentation were provided by NordicNeuroLab hardware (NordicNeuroLab, Norway). The data release is accompanied with a description of the paradigms, onset files, source code, and stimuli.

### Reliability

Our test-retest analysis has shown that most of the tasks provide reliable activation, which is defined as higher between session overlap than between subjects overlap, with the exception of overt verb generation and line bisection; the latter provides a particularly poor signal-to-noise ratio on a single-subject level. For more details of this analysis see [1].

### Scanning sequences

Data were acquired on a GE Signa HDxt 1.5 T scanner with an 8 channel phased-array head coil at the Brain Research Imaging Centre, University of Edinburgh, UK. The fMRI sessions used a different number of volumes depending on the task, but all sessions started with four dummy scans: (1) overt word repetition task, 76 volumes with sparse sampling (effective repetition time (TR) = 5 s, real TR = 2.5 s); (2) covert verb generation task, 173 volumes; (3) overt verb generation task, 88 volumes with sparse sampling (effective TR = 5 s, real TR = 2.5 s); (4) motor task, 184 volumes; (5) landmark task, 238 volumes. The order of the verb generation tasks were counterbalanced sequentially across subjects such that half of the subjects performed the task in the order [1 2 3 4 5], and the other half in the order [1 3 2 4 5]. All fMRI sessions employed a single-shot gradient-echo echo-planar imaging sequence with a field-of-view (FOV) = 256 × 256 mm, slice thickness 4 mm, 30 slices per volume, interleaved slices order, acquisition matrix 64 × 64, and TR = 2.5 s, flip angle = 90 degrees, and echo time (TE) = 50 ms. High resolution 3D T1-weighted volumes were acquired in the coronal plane with a FOV = 256 × 256 mm, slice thickness 1.3 mm, 156 slices, acquisition matri× 256 × 256, TR = 10s, TE = 4 s, and inversion time (TI) 500 ms. High angular resolution whole brain DTI scans were acquired with 64 directions (b = 1000 s/mm^2^; [6]) plus seven T2-weighted (b = 0 s/mm^2^) single-shot spin-echo echo-planar imaging volumes with a FOV = 256 × 256 mm, slice thickness 2 mm, 72 axial slices, acquisition matrix 128 × 128, TR = 16.5 s, and TE = 98 ms. Details of the gradient vectors and their strengths are provided in the data release. For a breakdown of the MR parameters see Table 1.

In summary, a test-retest task-based fMRI dataset is presented allowing researchers to investigate different processing methods and algorithms to improve reliability of brain measures. The utility of this dataset has been shown in previous reports where we have used it to investigate reliability and confounding factors in single subject fMRI [1], and to develop a new adaptive thresholding method that combines Gamma-Gaussian mixture modeling with topological thresholding to improve the reliability of cluster delineation [2]. Furthermore, the addition of high angular resolution DTI provides an opportunity to study the fusion between fMRI and DTI data such as in, for example, models of a dynamically changing network of activations (fMRI) constrained by anatomically derived structural connectivity, or models that attempt to define subsets of white matter fibers involved in a particular cognitive skill. Even though other publicly available test-retest datasets exist [7-9], they either include only one or two task-based fMRI sequences or are lacking DTI information. Therefore, to our knowledge there are no other publicly available test-retest datasets which provide five different task-based fMRI paradigms combined with the structural and DTI scans; thus making this dataset a unique resource for both neuroscientists and clinical researchers.

## Availability of supporting data

Each subject was assigned a random, unique identifier – using the DICOM confidential de-identification toolkit (http://sourceforge.net/projects/privacyguard/), this toolkit has replaced their name and any other medical identification information. DICOM files for each scanning sequence have been anonymized according to the Health Insurance Portability and Accountability Act guidelines. DICOM to NIfTI conversion was performed using the dcm2nii tool (http://www.mccauslandcenter.sc.edu/mricro/mricron/dcm2nii.html). To prevent visual identification, the 3D T1-weighted volumes have been defaced using mri_deface (http://www.na-mic.org/Wiki/index.php/Mbirn:_Defacer_for_structural_MRI – [10-12]). Therefore, seven NIfTI files are provided for each subject/session: five 4D fMRI, one 4D DTI, and one 3D T1-weighted volume scan.

Due to the fact that the overt language tasks were scanned using sparse sampling, we were able to record and analyze each subject’s responses. Due to privacy concerns these recordings cannot be included in this data release. This analysis lead to exclusion of one session of one subject of the overt word repetition task, due to the fact that the subject failed to perform the task correctly. Data and its description have been arranged according to the OpenfMRI (https://openfmri.org/) layout, and is available from the *GigaScience* Database [13].

## Abbreviations

fMRI: Functional magnetic resonance imaging; DTI: Diffusion tensor imaging; FOV: Field of view; TR: Repetition time; TE: Echo time.

## Competing interests

The authors declare that they have no competing interests.

## Authors’ contributions

KG acquired and analyzed the data, and drafted the manuscript; AS participated in the development of the thresholding algorithm and reliability analysis; MEB helped in developing the reliability analysis and in drafting the manuscript; IRW participated in the study design and coordination; JMW conceived the overall study and helped to draft the manuscript; CRP conceived the study, participated in its design and data analysis, and helped to draft the manuscript. All authors read and approved the final manuscript.
